# Evaluation of AA-CVD deposited phase pure polymorphs of SnS for thin films solar cells†

**DOI:** 10.1039/c9ra01938c

**Published:** 2019-05-16

**Authors:** Ibbi Y. Ahmet, Maxim Guc, Yudania Sánchez, Markus Neuschitzer, Victor Izquierdo-Roca, Edgardo Saucedo, Andrew L. Johnson

**Affiliations:** Department of Chemistry, Centre for Sustainable Chemical Technologies, University of Bath Bath BA2 7AY UK Ibrahim.Ahmet@helmholtz-berlin.de A.L.Johnson@bath.ac.uk; Catalonia Institute for Energy Research (IREC) Jardins de les Dones de Negre 1, pl. 2, 08930, St Adrià del Besòs Barcelona Spain

## Abstract

Six different thin film solar cells consisting of either orthorhombic (α-SnS) or cubic (π-SnS) tin(ii) sulfide absorber layers have been fabricated, characterized and evaluated. Absorber layers of either π-SnS or α-SnS were selectively deposited by temperature controlled Aerosol Assisted Chemical Vapor Deposition (AA-CVD) from a single source precursor. α-SnS and π-SnS layers were grown on molybdenum (Mo), Fluorine-doped Tin Oxide (FTO), and FTO coated with a thin amorphous-TiO_*x*_ layer (am-TiO_*x*_-FTO), which were shown to have significant impact on the growth rate and morphology of the as deposited thin films. Phase pure α-SnS and π-SnS thin films were characterized by X-ray diffraction analysis (XRD) and Raman spectroscopy (514.5 nm). Furthermore, a series of PV devices with an active area of 0.1 cm^2^ were subsequently fabricated using a CdS buffer layer, intrinsic ZnO (i-ZnO) as an insulator and Indium Tin Oxide (ITO) as a top contact. The highest solar conversion efficiency for the devices consisting of the α-SnS polymorph was achieved with Mo (*η* = 0.82%) or FTO (*η* = 0.88%) as the back contacts, with respective open-circuit voltages (*V*_oc_) of 0.135 and 0.144 V, and short-circuit current densities (*J*_sc_) of 12.96 and 12.78 mA cm^−2^. For the devices containing the π-SnS polymorph, the highest efficiencies were obtained with the am-TiO_*x*_-FTO (*η* = 0.41%) back contact, with a *V*_oc_ of 0.135 V, and *J*_sc_ of 5.40 mA cm^−2^. We show that mild post-fabrication hot plate annealing can improve the *J*_sc_, but can in most cases compromise the *V*_oc_. The effect of sequential annealing was monitored by solar conversion efficiency and external quantum efficiency (EQE) measurements.

## Abstract 2

Polymorph selective deposition of α- and π-SnS enables their evaluation as thin film PV absorber layers in various device structures.

## Graphical abstract

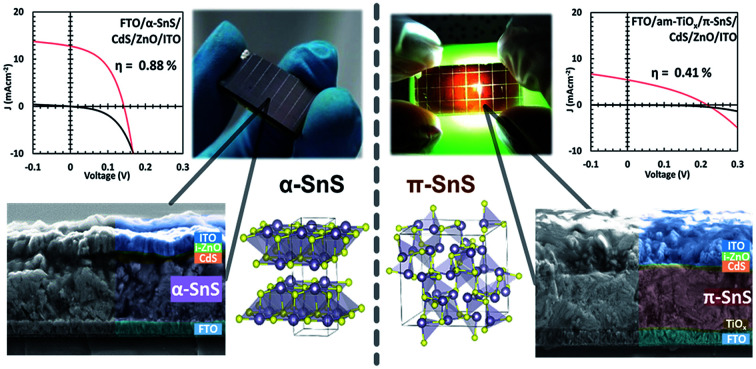

## Introduction

In recent years, considerable attention has been directed towards the study of tin mono-sulfide (SnS) as a possible earth abundant absorber material for thin film solar cells. It is well established that SnS can exist in a number of polymorphic phases, with early PV studies focused on the orthorhombic α-SnS phase as the most promising absorber layer for solar applications. Comparatively, less attention has been directed towards alternative polymorphic forms of SnS (*e.g.* π-SnS) and their application in the fabrication of PV cells.

### α-SnS thin film solar cells

**Fig. 1:**
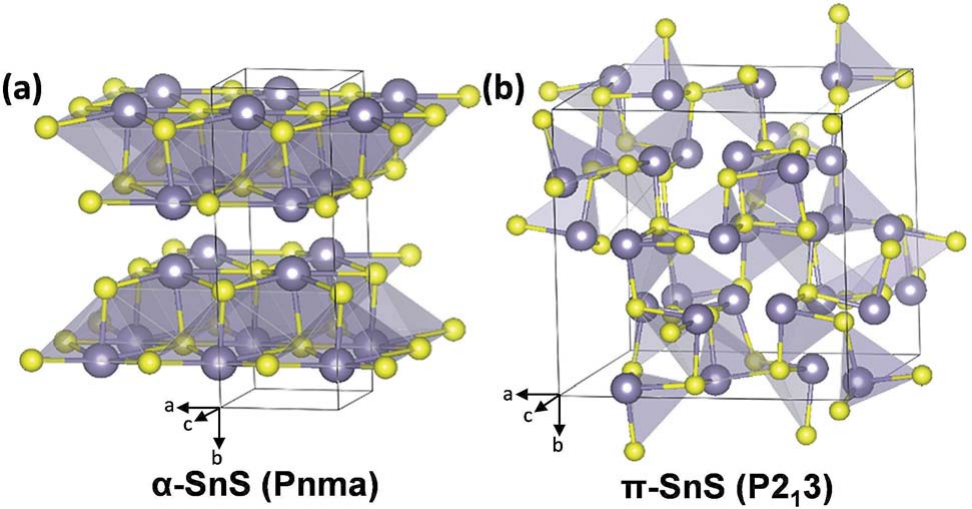
Tilted views of the assigned crystal structures and space groups for (a) the α-orthorhombic-SnS and (b) the π-cubic-SnS polymorph (Sn, grey; S, yellow).^32^

α-SnS, which is derived from the mineral herzenbergite, adopts an orthorhombic crystal structure (*Pnma* space group) with lattice parameters *a* = 11.44 Å, *b* = 4.03 Å and *c* = 4.40 Å (Fig. 1(a)). Appearing as a greyish-black material with a high absorption coefficient (>4 × 10^4^ cm^−1^, at wavelengths less than 800 nm), α-SnS is an intrinsic p-type semiconductor, with a direct band gap of ∼1.32 eV, and an indirect band gap of ∼1.1 eV.^1–4^ As such, these properties make α-SnS a promising material for thin film PVs. However, despite a theoretical Shockley–Queisser (S–Q) limit of ∼32%,^5^ the efficiencies of α-SnS solar cells fall consistently short of their theoretical maximum, with a current record efficiency of 4.4% (2014).^6^

Many studies have investigated the fabrication of α-SnS solar cells, *via* a wide range of deposition methods.^7,8^ The most recent advances in improving the solar cell performance have been focused on the optimization of a large range of material properties for each layer in the device structure. Essential improvements have focused on ensuring phase-pure α-SnS films are deposited, with large intercalated grains and minimal grain boundaries. The solid state structure of α-SnS consists of double-layers of SnS, which result in anisotropic physical, optical and electrical properties.^9,10^ The lack of control over this has been shown to have detrimental consequences on PV devices. The layered nature of α-SnS has also been attributed to inconsistent growth rates and the uncontrolled formation of SnS films with needle-, flake- or platelet-like morphologies.^4,11–17^ This poses a significant challenge for the fabrication of α-SnS thin film devices, since the morphological implications can result in a high level of grain boundary defects, pin holes, cracks, and an increased interfacial surface area at the p–n junction. All of these features result in higher recombination rates, device shunting or diminished light absorption.^18,19^

In an attempt to mitigate some of these negative features, processes such as H_2_S annealing of films have been trialed in order to increase grain size and lower grain boundary defects. Such procedures have been shown to control the hole concentration (3–5.7 × 10^15^ cm^−3^) in addition to increasing the charge carrier diffusion length. It has also been demonstrated that this treatment can decrease the number of sulfur vacancies (V_s_) in SnS films. These vacancies act as deep recombination centers and traps for electron minority carriers.^6,20^

Other device-enhancing processes have focused on the conduction band alignment of the SnS absorber and buffer layer. The use of a Zn(O_0.86_,S_0.14_) buffer layer has been proven to minimize conduction band offset (CBO). However, the electrical properties of the Zn(O,S) layer, in terms of carrier concentration, must be considered, and it has been shown that significant nitrogen doping is required to reduce the carrier concentration of this buffer layer in order to prevent device shunting.^6,20^

Intriguingly, prior to buffer layer deposition, surface oxidation of the α-SnS absorber layer has been shown to passivate the outer surface and improve the efficiency of completed devices. The simple process of exposing the SnS films to an atmosphere of air for 24 h can lead to significant surface oxidation, resulting in an enhancement of the solar conversion efficiency. It has been hypothesized that this step creates a diffusion barrier at the SnS/Zn(O,S) heterojunction, which either prevents sulfur loss, diffusion of Zn^2+^ ions across the junction or passivates trap states on the surface of α-SnS.^6,21^ All the aforementioned device optimization stages have been considered by the groups of T. Buonassisi and R. Gordon in order to achieve the current record α-SnS PV devices with verified 3.88 and 4.36% conversion efficiencies, where the α-SnS layer is deposited using either high vacuum evaporation (HVE) or atomic layer deposition (ALD) techniques, respectively.^6,21^

### π-SnS thin film solar cells

Phase pure nanocrystals,^22–24^ powders,^25^ and thin films of cubic π-SnS have all been shown to possess a wider direct band gap compared to α-SnS, ranging between 1.65–1.78 eV.^4,29–36^ Interestingly, there has been some debate over the assignment of the ‘cubic-SnS’ diffraction pattern to the correct crystal structure.^4,22–24,26–31^

It is now accepted that π-SnS consists of a large unit cell containing 64 atoms with an uncommon *P*2_1_3 space group and that π-SnS is the most accurate structural assignment for the ‘cubic SnS’ XRD pattern (Fig. 1(b)).^33^

The π-SnS structure is particularly interesting as it is non-centrosymmetric and exhibits chirality, which may be the origin of possible piezoelectric properties, as well the ability for enantiomerically pure samples to polarize light selectively.^22,23^ While π-SnS is considered thermodynamically metastable, experimental evidence has revealed that π-SnS is kinetically stable and requires heating (≥400 °C) for prolonged periods under either H_2_S^34^ or inert atmospheres^4^ to undergo conversion to the α-SnS ‘ground state’. Theoretical calculations have shown that π-SnS is only Δ*E* 1.76 kJ mol^−1^ (Popov *et al.*)^35^ or 2.19 kJ mol^−1^ (ref. 36) above the α-SnS ground state and thermodynamic global minimum. Furthermore, studies suggest that the π-SnS sits at a local potential-energy minimum, which is consistent with the observed kinetic stability.^4,37^

π-SnS is generally characterized as a p-type semiconductor, however previous studies have shown that π-SnS films are compensated^4^ or to have intrinsic hole charge carrier densities significantly smaller than the α-SnS films.^29^

Due to the high symmetry of the cubic unit cell of the π-SnS structure, it can be assumed that the growth rate of π-SnS crystals will be uniform across all axis/faces in comparison to the α-SnS polymorph. This feature can be observed by examining the morphologies of the majority of the π-SnS thin films deposited in this study and those deposited previously by chemical routes; they possess cubic like crystallites resulting in compact and uniform thin films, absent of pinholes, thus restricting the possibility of shunt pathways in full devices.^4,28^ A number of groups have reported the fabrication of thin film PV devices composed of π-SnS absorber layers.^27,28,34,38–40^ Even though the morphological features of the π-SnS films would be more appealing for fabricating single junction photovoltaic devices, the optical and electrical properties of the π-polymorph are suitable for a tandem system. With a direct band gap of 1.68–1.78 eV, π-SnS is potentially an ideal top layer absorber material for a tandem PV device, if π-SnS samples can be extensively optimized to generate a high photovoltage.^4,26–29,39^ Nair *et al.* calculated the maximum theoretical short circuit current density (*J*_sc_) for a device comprising of a single absorber layer of π-SnS (*E*_g_: 1.7) to be 21.7 mA cm^−2^, with a S–Q efficiency limit of ∼26%.^28^ Clearly, a π-SnS (∼1.7 eV) and α-SnS (∼1.1 eV) tandem would be a possibility and viable consideration if the performance of the individual PVs consisting of these absorber layers can be improved considerably. These attempts have been demonstrated by González-Flores *et al.* who have fabricated a series of α-SnS and π-SnS tandem devices in which they were able to achieve a solar conversion efficiency of 1.38% with a FTO/CdS/π-SnS-CUB/α-SnS/Carbon device structure. This tandem device results in a small improvement in solar conversion efficiency compared to the non-tandem α-SnS device.^41^

Table 1 presents the chronological progress of the research groups fabricating PV devices consisting of either mixed π-SnS/α-SnS or exclusively π-SnS or α-SnS absorber layers.

**Table 1:** An overview of the device parameters for selected PV devices composed of α-SnS or π-SnS absorber layers^a^

Device structure	Method of SnS deposition	*V*_oc_ (mV)	*J*_sc_ (mA cm^−2^)	FF (%)	Area (cm^−2^)	*η* (%)	Year [ref.]
**α-SnS**							
Mo/α-SnS/Zn(O,S)/ZnO/ITO	ALD	261	24.9	44	0.25	2.9	2014 [34]
Mo/α-SnS/SnO_2_/Zn(O,S):N/ZnO/ITO	ALD	372	20.2	58	0.23	4.38	2014 [6]
Mo/α-SnS/SnO_2_/Zn(O,S):N/ZnO/ITO	HVE	334	20.7	56	0.25	3.88	2014 [21]
Mo/α-SnS/CdS/i-ZnO/AZO	Vapor transport deposition	307	17.3	56	0.3	2.98	2018 [38]
FTO/TiO_2_/mp-TiO_2_/α-SnS/P3HT/PEDOT:PSS/Ag	Spin coating	313	24.8	39	0.1	3.01	2018 [42]
Mo/α-SnS/CdS/ZnO/AZO	Vapor transport deposition	342	19.8	58	0.2	3.93	2019 [19]
**A: Mo/α-SnS/CdS/i-ZnO/ITO**	**AA-CVD**	**135**	**12.96**	**47**	**0.1**	**0.82**	**This work**
**B: FTO/α-SnS/CdS/i-ZnO/ITO**	**AA-CVD**	**144**	**12.78**	**48**	**0.1**	**0.88**	**This work**
**C: FTO/am-TiO**_***x***_ **/α-SnS/CdS/i-ZnO/ITO**	**AA-CVD**	**85**	**3.74**	**31**	**0.1**	**0.1**	**This work**

**π-SnS**							
Ag/π-SnS/CdS/FTO	CBD	340	—	—	—	—	2008 [27]
Ag/(π + α)SnS/CdS/FTO	CBD	400	0.09	—	—	—	2008 [27]
Ag/α-SnS/π-SnS/CdS/FTO	CBD	370	1.23	44	0.04	0.2	2009 [39]
Mo/π-SnS/Zn(O,S)/ZnO/ITO	ALD	200	7.80	36	0.25	0.6	2015 [34]
SS/π-SnS/CdS/ZnO/AZO	CBD	470	6.23	44	1	1.28	2015 [28]
FTO/CdS/π-SnS/α-SnS/Carbon	CBD	488	6.96	41	0.2	1.38	2018 [41]
**D: Mo/π-SnS/CdS/i-ZnO/ITO**	**AA-CVD**	**133**	**5.96**	**27**	**0.1**	**0.21**	**This work**
**E: FTO/π-SnS/CdS/i-ZnO/ITO**	**AA-CVD**	**113**	**3.40**	**42**	**0.1**	**0.15**	**This work**
**F: FTO/am-TiOx/π-SnS/CdS/i-ZnO/ITO**	**AA-CVD**	**217**	**5.40**	**34**	**0.1**	**0.41**	**This work**

a FTO = fluorine doped tin oxide, ITO = indium tin oxide, AZO = aluminum doped zinc oxide, SS = stainless steel, mp-TiO_2_ = meso-porous TiO_2_, am-TiO_*x*_ = amorphous TiO_*x*_.

The current record conversion efficiency, using π-SnS, is 1.28%, in this case the principle absorber layer were deposited by chemical bath deposition (CBD) methods onto stainless steel substrates. Devices were completed with a CdS buffer layer, i-ZnO insulator and aluminum doped zinc oxide (AZO) top contact with an illumination area of 1 cm^2^.

The authors report that a main focus of improving the device efficiencies further would be by increasing the grain size of the ∼20 nm crystallites formed from low temperature methods, since this would improve carrier life time.^28^

It is clear that the polymorphism of SnS presents a very interesting case for investigation and there is still a lot to discover about the potential applications of both materials, which in turn maybe used to improve the device efficiencies of successive SnS, metal chalcogenide or tandem based solar cells.

## Experimental

### Molybdenum substrate preparation

Substrates of Mo coated soda-lime glass (Mo-glass) (800 nm, *R*_sq_ = 0.16 Ω sq^−1^) were deposited by DC-magnetron sputtering deposition (Ac450, Alliance Concepts, 4.3 W cm^−2^, 0.0006 mbar, 10 sccm Ar, 120 °C, 40 min) using 99.99% purity targets.

### Deposition of the 20 nm amorphous TiO_*x*_ seed layer onto TEC 7™-FTO (amTiO_*x*_-FTO)

The TiO_*x*_ precursor solution consisted of 35 μL of 2 M HCl in 2.5 mL of 2-propanol was added drop-wise to a solution with 370 μL titanium isopropoxide (97%, Sigma-Aldrich) in 2.5 mL of 2-propanol under heavy stirring. Cleaning was done by washing the TEC 7 FTO (NSG Ltd) substrates sequentially with a Hellmanex® solution (2 wt% in deionized water), acetone, ethanol and deionized water, and finally oxygen plasma treatment for 30 min. The TiO_*x*_ seed layer was deposited from the precursor solution of titanium isopropoxide *via* spin-coating at 2000 rpm for 60 s (2000 rpm s^−1^) then sequential annealing of the precursor thin-film at 150 °C and repeated 3 times.

### Selective aerosol assisted chemical vapor deposition of π-SnS or α-SnS thin films

Toluene solvents were dried using a commercially available solvent purification system (Innovative Technology Inc., Amesbury, MA, USA) and all solvents were degassed under argon prior to use. SnS thin films were deposited by Aerosol Assisted Chemical Vapor Deposition (AA-CVD) from a 0.08 M toluene solution of the well-defined single source precursor (1), (dimethylamido)(*N*-phenyl-*N*′,*N*′-dimethylthiouriate)tin(ii) dimer/Sn(ii) thio-ureide complex reported previously (see Scheme S1†).^4^ Either borosilicate glass, Mo-glass, FTO (TEC 7) or TiO_*x*_-FTO (TEC 7) substrates were placed inside the quartz reactor tube/deposition chamber. The deposition chamber was purged with argon gas prior deposition. The aerosol was generated using a TSI 3076 constant output atomizer. The argon gas flow was monitored *via* a bubbler and gas pressure fixed at 5 bar until it reached the atomizer. Further details can be found in previous reports.^4^ π-SnS films were deposited at 300 °C for 100 min and α-SnS films were deposited at 375 °C for 90 min, then at 400 °C for 10 min to prevent the formation of secondary π-SnS phases at cooler regions of the deposition chamber. After deposition the samples were cooled to room temperature under argon atmosphere and stored in air. UV-Vis transreflectance measurements of the α-SnS and π-SnS deposited onto glass were performed using a UV-VIS Lambda 950 spectrophotometer (PerkinElmer®) equipped with an integrating sphere and center mount.

### Device completion

Prior to CdS deposition the surface of the SnS absorber layers were etched by submerging films in a 10% aqueous solution of (NH_4_)_2_S for 30 s and then rinsed with deionized water. This step was used to clean the surface of the SnS, by removing a surface layer of SnS and exposing an underlying surface. A ∼50 nm CdS buffer layer was deposited onto the SnS absorber layer following procedures published by M. Neuschitzer *et al.*,^43^ where absorber layers are submerged in an aqueous pH = 9.7 ammonium hydroxide chemical bath solution, which is stirred, and treated with 0.12 M CdSO_4_ and 0.3 M thiourea at 70 °C for 7 min. After this time the devices are removed from the solution and washed several times with deionized water and dried under a fast stream of N_2_. The deposition of the i-ZnO (10 nm) layer and 200 nm ITO top contact was achieved by DC-sputtering techniques a RT (*R*_sq_ = 10 Ω sq^−1^) using a CT100, Alliance Concepts. All devices had a cell active area of 0.1 cm^2^, which was produced by using a microdiamond scriber MR200 OEG (OEG Gesellschaft für Optik, Elektronik & Gerätetechnik mbH, Frankfurt, Germany) with a scribed line width of ∼20 μm. The back contact was electrically connected by soldering indium contacts onto an exposed area of the Mo or FTO created by mechanical abrasion at the edge of the devices. Annealing treatment of the completed devices were performed in air using a calibrated hot plate, and following similar procedures reported elsewhere.^44^

### Efficiency measurements

Dark and illuminated *J*–*V* curves were measured using a Sun 3000 class AAA solar simulator (ABET Technologies Inc., Milford, Connecticut, USA; uniform illumination area of 15 × 15 cm), starting from negative to positive voltages. Measurements were carried out at 25 °C, and before measurements, the intensity of the solar simulator was calibrated to 1 sun AM 1.5 using a Si reference cell.

### External quantum efficiency (EQE) measurements

The measurement of the External Quantum Efficiency (EQE) *vs.* photon energy of completed devices were measured with a pre-calibrated Bentham PVE300 system in the 300–1200 nm wavelength range, and calibrated using Si and Ge photodiodes. Reversed voltage-biased EQE curves were collected by connecting a Keithley 2400 source meter (Keithley Instruments Inc., Cleveland, Ohio, USA) directly to the primary coil of the transformer and biasing the device at the desired potential, referenced against the cell voltage.

### Materials characterization

Morphological characterization and cross section analysis was undertaken with field emission scanning electron microscopy (FESEM) through a Zeiss series Auriga microscope, using an accelerating voltage of 5 kV. X-ray diffraction data were collected using a BRUKER D8-Advance analyzer. The X-ray diffraction patterns were collected for the thin films using the flat plate mode with a Bragg–Brentano geometry from 5 to 70° at 2° min^−1^. X-rays were generated from a Cu source at wavelengths of 1.54 Å. Raman scattering spectra were measured with Horiba Jobin-Yvon T6400 spectrometer coupled with a CCD detector in a backscattering configuration though the Olympus objective. A 514.5 nm Ag^+^ laser was used for the spectra excitation, and the laser energy density did not exceed 30 kW cm^−2^, which excluded any influence to the sample surface (heating, decomposition, evaporation) during the measurements. The Raman spectra has been corrected imposing the position of main Si peak of the monocrystalline sample to 520 cm^−1^. The gas laser as excitation source and a high resolution triple monochromator allowed to reveal the Raman modes of the different SnS polymorphs in the low wavenumber range, starting from 20 cm^−1^.

## Results and discussion

### Characterization of α-SnS and π-SnS thin films

This study takes advantage of the previously reported method of producing either α- or π-SnS thin films from AA-CVD and using the well-defined Sn(ii) thio-ureide complex (1) as the single source precursor.^4^ The molecular structure of 1 is provided in the ESI.† The initial intention of this study was to further characterize and compare how the growth and morphology of different SnS polymorphs can be affected by changing the substrate/back contacts they are deposited on. In this study we have selectively deposited α-SnS or π-SnS onto three different substrates, consisting of molybdenum coated soda-lime glass (Mo-glass), blank TEC 7™ FTO (FTO) or TEC7™ FTO coated with a ∼20 nm amorphous TiO_*x*_ (TiO_*x*_-FTO) layer.

### X-ray diffraction analysis

**Fig. 2:**
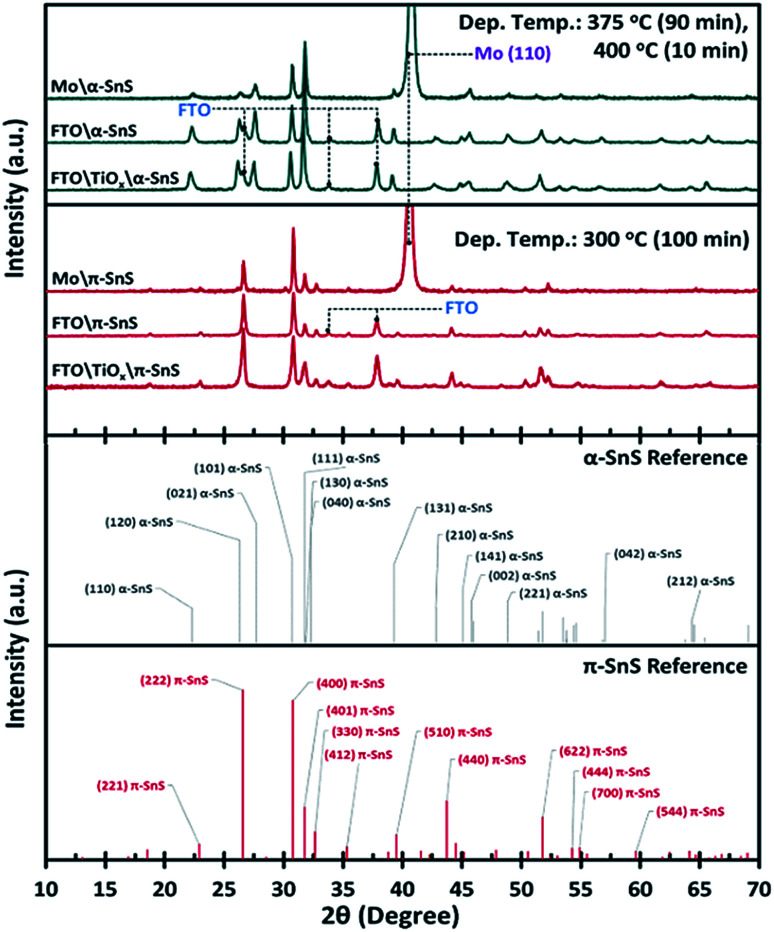
Comparison of XRD patterns, with assigned reference peaks, for selectively deposited α- and π-SnS thin films deposited onto Mo, FTO or am-TiO_*x*_/FTO substrates by AA-CVD using the Sn(ii) thio-ureide complex as a single source precursor.

To confirm the phase purity of the α-SnS and π-SnS absorber layers on different substrates, XRD analysis was performed on each 40 × 20 mm sample. It is clear from the XRD analysis (Fig. 2) that we can selectively grow phase pure films of either α-SnS or π-SnS on all substrates, where the polymorph selectivity is dependent on the temperature at which the AA-CVD deposition is undertaken. The SnS films deposited at a higher temperature range (at 375 °C for 90 min, then at 400 °C for 10 min), shows the presence of diffraction peak positions and relative intensities consistent with the formation of orthorhombic α-SnS (JCPDS no. 00-039-0354,^45^ PDF 39-0354 (ref. 46)) and in accordance with previous reports.^27^ The unit cell parameters, *a* = 4.33 Å, *b* = 11.20 Å, *c* = 3.98 Å, determined for these samples match that of the known orthorhombic SnS crystal structure.^46^ The XRD analyses of all α-SnS films either grown on Mo, FTO or am-TiO_*x*_ show no preferential orientation, such that all diffraction peaks present intensities relative to those predicted for poly-crystalline samples.

Using the known *hkl* values and the associated peak positions for the reported π-SnS structure, an average unit cell constant of *a* = 11.6 Å was determined for these π-SnS thin film samples. This value is consistent with that reported for other π-SnS thin films^29^ and powders,^22^ and is well matched with the unit cell constant (*a* = 11.595 Å) of the calculated crystal structure determined by computational methods.^33^

Since the XRD analyses was executed using a Bragg–Brentano geometry, diffraction peaks corresponding to FTO and Mo substrates are also observed due to the X-ray penetration depth. Furthermore no peaks assigned to either the anatase or rutile TiO_2_ crystal structure were observed providing evidence that the TiO_*x*_ layer is amorphous.

### Raman spectroscopy

**Fig. 3:**
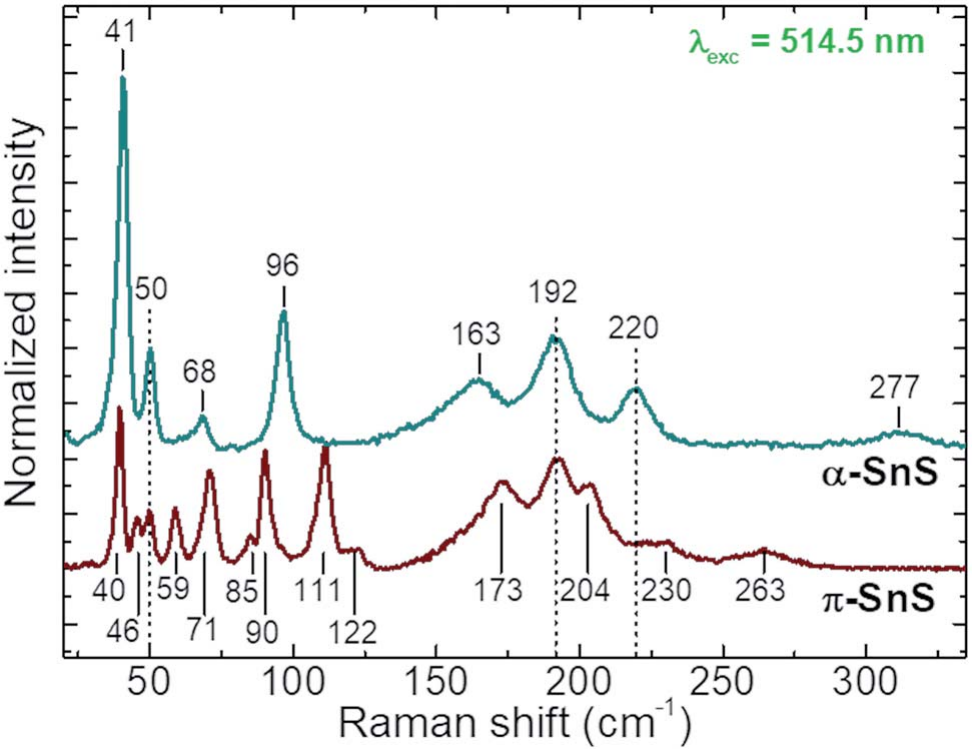
Comparison of Raman spectra for α-SnS and π-SnS samples deposited onto FTO substrates, using a 514.5 nm laser wavelength. For convenience, the spectra were normalized to the peak at 192 cm^−1^ and shifted along the *Y* axis.

To date only a few reports have examined the Raman scattering spectra of the different SnS polymorphs,^4,22,47–49^ and no strict distinctions in their spectra have been presented so far. In the present study in the Raman spectra of the α-SnS and π-SnS thin films allowed for the resolution of 8 and 15 peaks, respectively (Fig. 3).

The detected peaks closely match the previously reported experimental Raman spectra,^4,22,47–49^ as well as theoretically calculated vibrational mode positions.^36^ An evident difference between the Raman spectra of two polymorphs has been observed. However, broadening of the peaks in the range 150–250 cm^−1^ make it difficult to be used for clear polymorph identification. On the other hand, the low spectral range (<120 cm^−1^), with intense and sharp peaks, is much more attractive from the point of view of SnS polymorph identification. The detection of the peak at 96 cm^−1^ allows the presence of α-SnS to be confirmed, while the peak at 111 cm^−1^ is attributed to the presence of π-SnS. Relative intensity quantification of these two peaks can be used to evaluate the relative ratio of the α- and π-SnS phases.

### Characterization of α-SnS and π-SnS solar cells

**Fig. 4:**
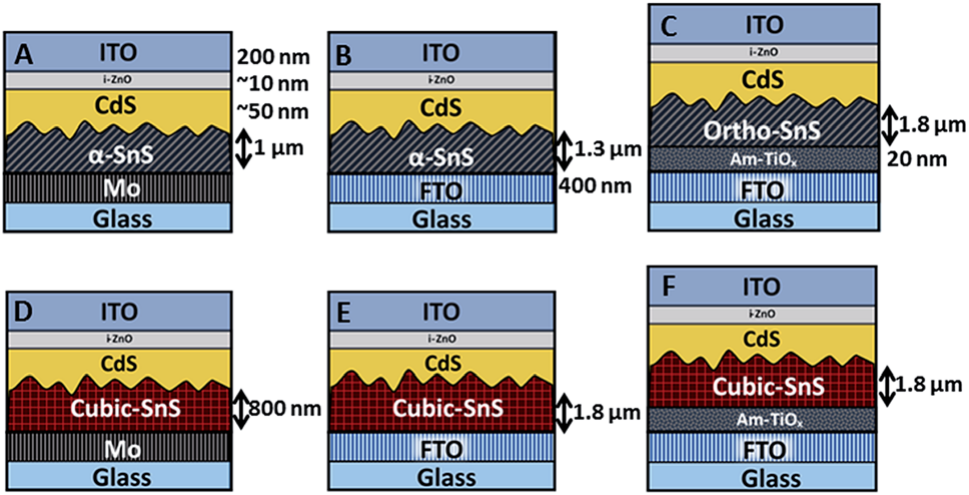
Illustration of the six devices investigated in this study. Layer thicknesses are indicated and same across all devices unless specified.

It was of interest to observe how varying the back contact can affect the growth of the SnS absorber layer, and furthermore the performance of the completed PV devices. Once the SnS layers were deposited on the three substrate types, the devices were subsequently completed using the same deposition methods. Firstly a ∼ 50 nm n-type CdS buffer layer was deposited onto the SnS by CBD, then a ∼10 nm i-ZnO layer and a 200 nm ITO layer was applied *via* RF sputtering. Resultantly, six different device arrays, listed from A to F, were fabricated and investigated in this study, as shown in Fig. 4.

### Effects of substrate and polymorph type of SnS grain size, morphology, and film thickness

**Fig. 5:**
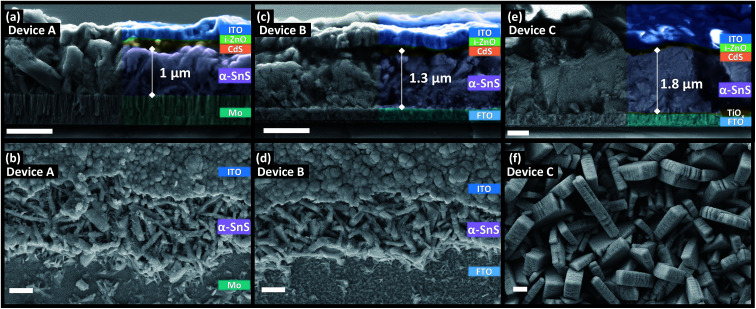
Cross section (top) and top down (bottom) SEM micrographs of α-SnS PV devices A, B, and C after hot plate annealing. Top down images of devices A and B show regions of the device where device layers have been purposely removed to expose underlying films. Scale bar = 1 μm.

**Fig. 6:**
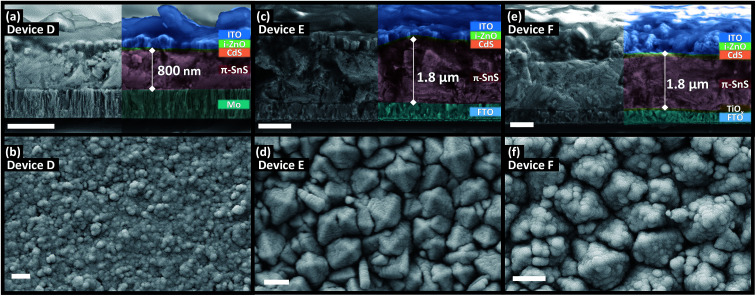
Cross section (top) and top down (bottom) SEM micrographs of π-SnS PV devices D, E, and F after hot plate annealing. Scale bar = 1 μm.

Fig. 5 and 6 shows the top down and cross section SEM images of devices A, B, C, D, E, and F. There appears to be a strong substrate dependence on the growth rate and morphology of both the α-SnS and π-SnS crystallites. Considering all films were deposit for the same total duration (100 min), the films thicknesses vary considerably and depends on the substrate and SnS polymorph. It is clear that film growth rates are much higher for films grown on FTO and am-TiO_*x*_ compared to Mo.

The impact of substrate on the film growth rate of films produced from CVD methods has been widely documented;^38,50–53^ in this case the higher growth rate of SnS on FTO and am-TiO_*x*_ could be a contribution of their higher surface roughness, but could also be due to the surface chemistry of metal oxides.

The surfaces of Mo, FTO and TiO_*x*_ layers differentiate significantly particularly in the surface roughness, surface chemistry and density of nucleation sites. The high surface roughness as well as the hydroxylated surfaces of FTO and TiO_*x*_ will have an increased number of nucleation sites as compare to the Mo surfaces. Particularly in non-equilibrium conditions and at low deposition temperatures the concentration of nucleation sites will strongly influence the growth rate. Indeed faster film growth rates are observed on FTO and TiO_*x*_ substrates.^54^

From Fig. 5 it is clear that the α-SnS crystallite morphologies are strongly affected by the substrate surface. For the Mo and FTO substrates the grains form small plate like morphologies with many grain boundaries. Comparatively, α-SnS growth on FTO treated with amorphous TiO_*x*_, consists of larger plate like grains with much more pronounced growth in the perpendicular domain of the crystallite plates. These plate like features are often observed in other reports of α-SnS thin films deposited by similar methods.^55–57^ It is known that these morphologies evolve from the anisotropic crystal structure of α-SnS, which is indeed a layered system, and therefore directs the isotropic growth of the crystallite grains.

There is a clear distinction between the crystallite morphologies of the α-SnS and π-SnS phase (see Fig. 5 and 6), which has also been observed previously.^4,58^ As mentioned the π-SnS cubic polymorph is known to grow isotropically and crystallite grains are reported to form cube like morphologies, influenced by the isotropic nature of the cubic π-SnS crystal structure.^32^ SEM micrographs in Fig. 6 shows that indeed compact cube like crystallite morphologies are formed for all thin film samples of π-SnS deposited by AA-CVD. Interestingly for the π-SnS polymorph grown on Mo substrates the grains are small ranging from 100–300 nm compared to the same films grown on FTO and am-TiO_*x*_-FTO substrates, where the samples show much larger grain growth ranging from 700–1000 nm in size. A hypothesis for this observation may be that the metal oxide surfaces of FTO and am-TiO_*x*_-FTO match well with the lattice structure of the π-SnS polymorph compared to Mo.

It is important to consider the morphologies of all SnS samples as this property of SnS films is known to be a significant factor that influences the performance of SnS based photovoltaics. Films with large compact grains can minimize grain boundaries and shunt pathways, which sequentially diminishes the device's rectifying behavior. For these reasons, it is clear that the morphologies of all α-SnS are less than ideal, as compared to the π-SnS samples.

### Device performance and effects of post hot plate annealing of SnS PV devices

**Fig. 7:**
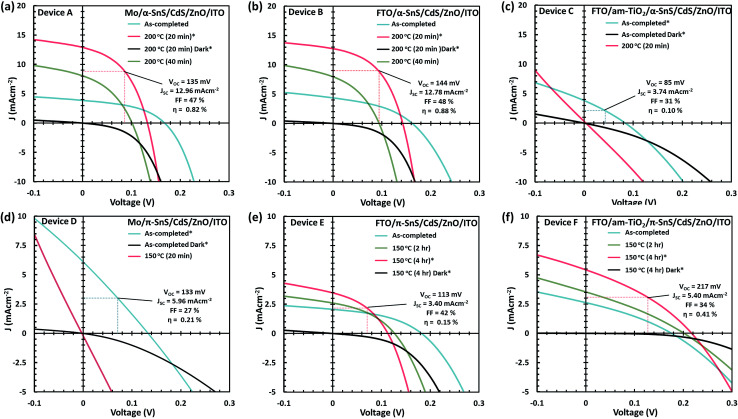
*J*–*V* characteristics in the dark and under simulated AM 1.5 G illumination for the as-completed and after subsequent hot plate treatment of the solar cells A–F (a–f). Only the PV device parameters, for the highest pixel efficiencies and optimized conditions, are presented.

**Fig. 8:**
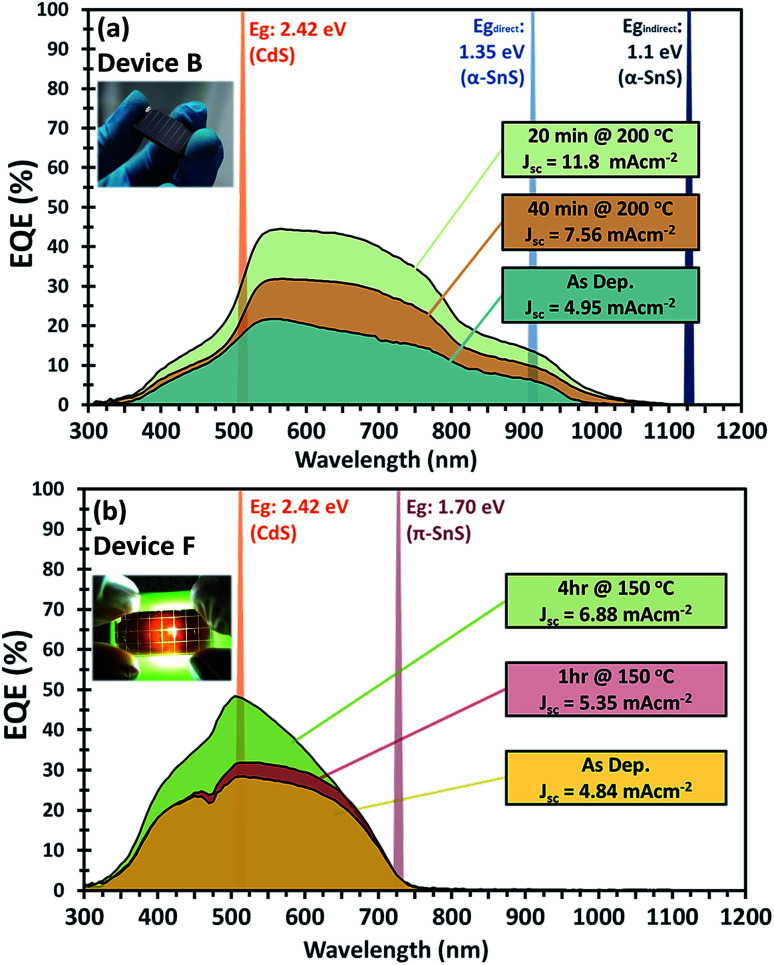
External quantum efficiency (EQE) *vs.* photon wavelength for (a) device B consisting of FTO/α-SnS/CdS/ZnO/ITO and (b) device F consisting of FTO/am-TiO_*x*_/π-SnS/CdS/ZnO/ITO. The EQE measurements after consecutive hot plate annealing treatments are presented. Inset are photographic images of each device respectively.

*J*–*V* characteristics and optimized device parameters of the various SnS solar cells are presented in Fig. 7. Solar conversion efficiencies were measured for all pixels within as-deposited samples and summarized in Table S1 and Fig. S1.† Highest efficiency pixels were selected and devices were further optimized by post deposition hot plate annealing. A summary of all the PV device parameters for the optimized post deposition hot plate annealing steps are presented in Table S2.† All devices were annealed at 150 or 200 °C in air for successive annealing times. As can be seen from the *J*–*V* characteristics of all devices post deposition hot plate annealing significantly affects the *J*_sc_. In order to closely monitor these effects after each annealing step, an EQE *vs.* photon wavelength plot was measured for each device at short circuit potential (see Fig. 8 and ESI Fig. S2 and S3†).

For devices containing the α-SnS polymorph (A, B, and C) hot plate treatment at 150 °C for 20 min, 40 min, 1 hour and 4 hours had no significant effect on the device performance. However, using a hot plate temperature of 200 °C and an annealing time of 20 minutes, device efficiencies for A and B improved significantly (see Fig. 7(a and b)). Furthermore, from the *J*–*V* characteristics (Fig. 7(a and b)) and from the EQE spectra of devices A and B, shown in Fig. 8(a) and S2,† this improvement is attributed to a significant increase in the *J*_sc_, whilst the *V*_oc_ is slightly compromised. Specifically looking at the change in the EQE plot of device B (Fig. 8(a)), it is obvious that hot plate annealing at 200 °C for 20 minutes, can improve the current extraction and the EQE increases at all photon energies. However, prolonged annealing at 200 °C has detrimental effects on the device performance, and the solar conversion efficiencies and EQEs decreased for both α-SnS devices A and B after 40 minutes of annealing (Fig. 8 and S2†). Eventually, the devices became completely shunted after 1 hour of annealing at 200 °C. The *J*–*V* character for device C (as-completed) showed a significantly small solar conversion efficiency and *V*_oc_ compared to the other α-SnS devices A and B. As can be seen from Fig. 7(c), in contrast to devices A and B, device C becomes completely shunted after hot plate treatment at 200 °C for 20 min. The reason for this observation could be due to the large granular morphology of the α-SnS grown on amorphous TiO_*x*_. We observe from the SEM images of device (Fig. 5(e and f)) that the α-SnS grains are significantly larger and rough, when compared to those in devices A and B. These larger grains do not allow for a uniform deposition of the buffer layers and top contact and thus promotes the formation of pinholes. As is reported elsewhere,^19^ it is expected that these morphological features can assist in the formation shunt pathways, which can be further promoted after hot plate annealing.

For devices containing the π-SnS polymorph, hot plate treatment at 200 °C was significantly detrimental to the device performance at any annealing time from 20 min to 4 h and all devices decreased in efficiency and were completely shunted after 40 minutes. However, annealing at 150 °C would improve the device efficiencies, and similarly, hot plate annealing particularly increased the *J*_sc_. Interestingly, prolonged annealing at 150 °C, for an extended period of 4 hours noticeably improved the device performance of E and F. Furthermore, the EQE across all photon energies increased with each period of annealing (see Fig. 7(e, f), 8(b), and ESI Fig. S2†). After 4 hours of annealing at 150 °C there was no apparent increase in these device efficiencies nor a substantial increase in EQE across all photon energies. As completed device D (Fig. 7(d)) showed limited performance and showed no rectifying properties after annealing at 150 °C for 20 min. The film thickness of the π-SnS layer in device D was significantly smaller (∼800 nm) as compared to E and F (∼1.8 μm). Therefore, for the similar reasons mentioned above, it can be assumed that the smaller thickness of the π-SnS layer in device D may promote the formation for shunt pathways, which is evident from the *J*–*V* curves of device D after initial heat treatment (Fig. 7(d)).

There are a number reasons why post fabrication annealing can improve the device performance and increase the photocurrent collection. The same effect has been observed in other type's thin film photovoltaic devices based on Copper Indium Gallium Selenide (CIGS), Copper Zinc Tin Sulfur/Selenides (CZTS/Se) and Copper Tin Sulfide (Cu_2_SnS_3_) absorber layers.^59–62^

Electron beam-induced current (EBIC) analysis of CIGS devices have shown there is a significant increase in the space charge region (SCR) at the hetero junction after annealing complete devices with a CIGSSe/CdS p–n junction. An increase in the SCR implies a decrease in the density of carriers and will assist the degree of carrier separation at the junction and therefore increase the EQE. However, a large SCR will result in a lower built-in potential, and thus resulting in a decrease in *V*_oc_.^61^ Both these changes in the *J*–*V* parameters are also witnessed after post annealing the SnS devices within this study.

Examination of the EQE spectra for device F with the FTO/am-TiO_*x*_/π-SnS/i-ZnO/ITO structure, it is clear to see the effect of hot plate annealing has on the profile of photocurrent extraction (see Fig. 8(b)). There is a distinctive increase in EQE with each annealing step. The as-completed device reached a maximum EQE of 30% at 510 nm and after 4 hours of annealing, we observe an increase to a maximum of 50% at the same wavelength. For further examination of device F, we measured the EQE spectrum at a reverse bias below the short circuit potential. By doing this it is possible to extend the degree of band bending at the p–n junction and subsequently increase the space charge region. Measuring the EQE spectrum of the device at different reverse bias, we can determine the maximum amount of photo-generated charge carriers that can be extracted from the absorber layer if the p–n junction can be modified to maximize the space charge region during device operation. Fig. S4† presents the EQE profile of device F under various reverse bias from 0 to +2 V. From the different EQE measurements of device F (after hot plate treatment) we observe a larger extraction of photocurrent up to a reverse bias of +2 V, where the EQE spectrum saturates, thus charge carrier extraction reaches a limit, and the EQE reaches a maximum of ∼66% at 510 nm. By integrating across the photon flux of an AM 1.5 G spectrum with the EQE profile measured at a bias potential +2 V, it is calculated, under these band bending conditions, a photo generated current density of ∼11 mA cm^−2^ could be generated from these π-SnS absorber layers, which constitutes to 45% of the maximum theoretical photocurrent density for π-SnS.^28^

Respective band gaps have been determined from thin film samples of each SnS polymorph by UV-Vis-NIR diffuse transflectance measurements (see Fig S5†). The differences in the optical band gap of the two polymorphs, has a clear influence on the profile of the EQE spectra for respective devices consisting of α- and π-SnS absorber layers. Subsequently, there are a number of reported methods for determining the band gap of absorber layers within completed devices. In order to make an objective comparison between PV devices often the PV bandgap (*E*^PV^_g_) is calculated from the EQE data and reported.^63–65^

*E* ^PV^_g_ is given by:1
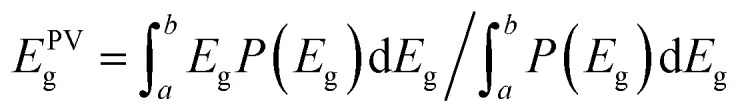
where *P*(*E*_g_) = d/d*E* EQE(*E*) is the probability distribution function for the distribution of Shockley–Queisser-type bandgap energies and *E* is the photon energies. The maximum of d/d*E* EQE gives an estimate for *E*^PV^_g_, if *P*(*E*_g_) is approximately Gaussian.^65^

From Fig. S6,† an *E*^PV^_g_ of 1.3 eV is determined for devices using α-SnS as the absorber layer (devices A, B, and C). This *E*^PV^_g_ energy is comparable to the direct band gaps energies calculated from optical measurements of α-SnS samples (*E*^direct^_g_ = 1.28 eV) deposited onto glass from the same method and precursor (see ESI Fig. S5(b)†).

For devices using π-SnS (devices D, E, and F), the *E*^PV^_g_ varies between 1.75–1.79 eV (Fig. S7†). These values are similar to the direct band gap (*E*^direct^_g_ = 1.7 eV) determined by optical measurements of π-SnS thin film samples (see Fig. S5(b)†). Interestingly, the EQE data obtained from these SnS solar cells, of different polymorphs, closely match the EQE data measured by photo-electrochemical methods for SnS photo-electrodes of different polymorphs. In this previous report, samples of α-SnS and π-SnS were deposited by AA-CVD from the same precursor onto Mo substrates.^4^

For devices C and F it is expected that by depositing an n-type TiO_*x*_ layer onto the surface of the FTO substrate would block holes from reaching the FTO back contact, and therefore impose a large series resistance. However, the TiO_*x*_ layer in this case has been deposited onto the FTO at low temperatures (150 °C), and it is expected to be porous and amorphous. It is assumed the TiO_*x*_ layer contains a high concentration of defects since it is in an amorphous state. This type of amorphous TiO_*x*_ has been reported to act as a hole conductor.^66^

Indeed for both devices C and F we observe photocurrents which indicate that the amorphous TiO_*x*_ layer does not act as a significant hole blocking layer.

Most interestingly is that device F was the best PV device composed of a π-SnS absorber layer, after post deposition hot plate annealing. The higher performance of this particular device is attributed to the improved crystallite growth and larger grain size of π-SnS absorber layers, which appears to be induced by the TiO_*x*_ seed layer on the FTO substrate. In relation to this small crystallite size of the π-SnS absorber layers have previously been attributed to limiting the efficiency of such PV devices.^41^

Comparing the performance of the best α-SnS (A and B) and π-SnS (E and F) solar cells (Fig. 7), the *J*_sc_ for the α-SnS devices are significantly larger. The higher photocurrents is likely to be due to the narrower band gap of this polymorph, thus the spectral range for photocurrent generation is larger as is obvious from the EQE measurements (Fig. 8). Interestingly the *V*_oc_ for the best π-SnS device, device F, is 217 mV and comparatively larger than the *V*_oc_ for the best α-SnS device, 144 mV. The larger *V*_oc_ can be due to the larger band gap of this polymorph, and assuming the percentage of free energy losses in both SnS absorber layers are the same, a larger band gap semiconductor can theoretically produce a larger photo-voltage. However, for all devices the *V*_oc_ is still significantly small in comparison to the band gap of both α-SnS and π-SnS, and less than other reports of SnS solar cells.^6,21,27,28,34,38,39,41,42^ Significantly higher *V*_oc_ values have been achieved in recent reports for devices consisting of an α-SnS/π-SnS tandem absorber layer, reaching 488 mV.^41^

## Conclusions

This work shows the first demonstration of α-SnS and π-SnS solar cells fabricated by AA-CVD using a single source precursor. In addition, we present the fingerprint Raman spectra from a 514.5 nm excitation wavelength for the two analyzed polymorphs of SnS, which highlight features for the clear distinction between the α- and π-polymorphs by means of Raman spectroscopy. Particularly from this type of vapor deposition method, we show how the substrate type and its surface chemistry can strongly impact the growth of SnS thin films consisting of either polymorph. We subsequently report the performance of 6 solar cell structures; consisting of a CdS buffer layer, α-SnS or π-SnS absorber layers and Mo, FTO or FTO/am-TiO_*x*_ back contacts. Notably a systematic method of improving and optimizing SnS PV devices by mild post hot plate annealing it documented. Only α-SnS devices with a Mo and FTO back contact and π-SnS devices with FTO and FTO/am-TiO_*x*_ back contacts maintained rectifying behavior after hot plate annealing, which is a result of the distinctly different crystallite growth and morphologies for these two polymorphs on the three types of substrates varied in this study. It is shown that post fabrication hot plate annealing can significantly improve the *J*_sc_ for these devices reported. The highest solar conversion efficiencies consisting of α-SnS and π-SnS absorber layers were 0.88% (FTO/α-SnS/CdS/i-ZnO/ITO) and 0.47% (FTO/am-TiO_*x*_/π-SnS/CdS/i-ZnO/ITO), respectively. The successful growth of π-SnS onto FTO and amorphous TiO_*x*_ surfaces establishes the possibility that this wide band gap polymorph (*E*_g_: ∼1.7 eV), with future optimization, has the potential to act as a top absorber layer in tandem PV devices.

## Conflicts of interest

There are no conflicts to declare.

## Supplementary Material

RA-009-C9RA01938C-s001
