# Supplementary material for: Evaluation of AA-CVD deposited phase pure polymorphs of SnS for thin films solar cells
Source: RSC Adv. 2019 May 16;9(26):14899–909. doi: 10.1039/c9ra01938c (PMC9064236; doi:10.1039/c9ra01938c)
Supplement: RA-009-C9RA01938C-s001 [file RA-009-C9RA01938C-s001.pdf]

Electronic Supplementary Material (ESI) for RSC Advances.
This journal is © The Royal Society of Chemistry 2019

Supporting Information

Evaluation of AA-CVD Deposited Phase Pure Polymorphs of SnS for Thin Films Solar Cells

Ibbi Y. Ahmet[a]*, Maxim Guc[b], Yudania Sánchez[b], Markus Neuschitzer[b], Victor Izquierdo-Roca[b], Edgardo Saucedo[b], and Andrew L. Johnson[a]*

[a.]Department of Chemistry and Centre for Sustainable Chemical Technologies, University of Bath, Bath BA2 7AY, United Kingdom. Email: Ibrahim.ahmet@helmholtz-berlin.de, A.L.Johnson@bath.ac.uk

[b.]Catalonia Institute for Energy Research (IREC), Jardins de les Dones de Negre 1, pl.2, 08930, St Adrià del Besòs, Barcelona, Spain.

Table S1: Summary of functional and shunted pixels per sample for as-deposited α-SnS and π-SnS PV devices.

Device Structure	Treatment	No. of Shunted Pixels	Number of Rectifying Pixels
**α-SnS**			
A1: Mo/α-SnS /CdS/i-ZnO/ITO	As completed	1	5
A2: Mo/α-SnS /CdS/i-ZnO/ITO	As completed	15	6
B1: FTO/α-SnS/CdS/i-ZnO/ITO	As completed	2	8
B2: FTO/α-SnS/CdS/i-ZnO/ITO	As completed	10	6
C1: FTO/am-TiO_x/α-SnS/CdS/i-ZnO/ITO	As completed	8	2
C2: FTO/am-TiO_x/α-SnS/CdS/i-ZnO/ITO	As completed	11	2
**π-SnS**			
D1: Mo/π-SnS/CdS/i-ZnO/ITO	As completed	1	5
D2: Mo/π-SnS/CdS/i-ZnO/ITO	As completed	11	9
E1: FTO/π-SnS/CdS/i-ZnO/ITO	As completed	9	6
E2: FTO/π-SnS/CdS/i-ZnO/ITO	As completed	0	10
F1: FTO/am-TiOx/π-SnS/CdS/i-ZnO/ITO	As completed	0	6
F2: FTO/am-TiOx/π-SnS/CdS/i-ZnO/ITO	As completed	9	9

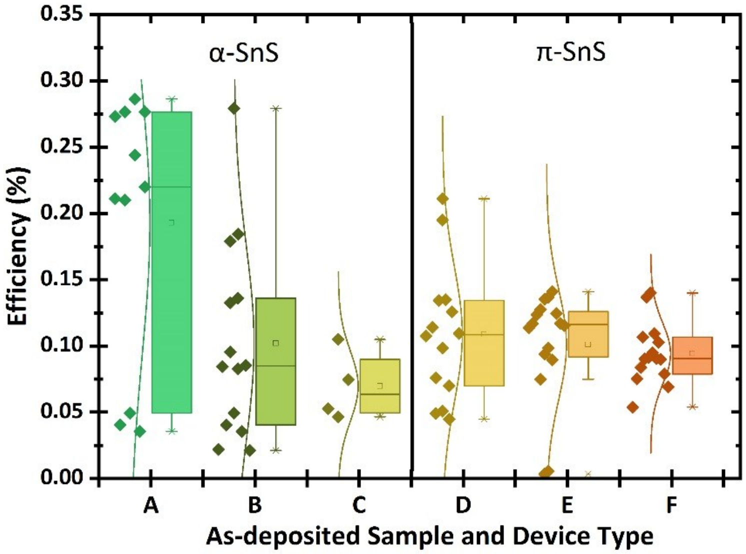

Fig. S1 Statistical box plot for the distribution of solar conversion efficiencies for individual pixels within as-deposited samples consisting of device structures A, B, C, D, E, and F.

Table S2: Summary of all measured α-SnS and π-SnS PV devices under an AM 1.5G illumination with different post fabrication hot plate annealing treatments.

Device Structure	Post Fabrication Hot Plate Annealing Method	V_{OC} (mV)	Jsc (mA cm-2)	FF (%)	Area (cm^{-2})	η (%)
**α-SnS**						
A: Mo/α-SnS /CdS/i-ZnO/ITO	As completed	170	3.81	45	0.1	0.29
**A: Mo/α-SnS /CdS/i-ZnO/ITO**	**200 ^{o}C, 20 min**	**135**	**12.96**	**47**	**0.1**	**0.82**
A: Mo/α-SnS /CdS/i-ZnO/ITO	200 ^{o}C, 40 min	100	8.01	43	0.1	0.35
B: FTO/α-SnS/CdS/i-ZnO/ITO	As completed	160	4.26	40	0.1	0.28
**B: FTO/α-SnS/CdS/i-ZnO/ITO**	**200 ^{o}C, 20 min**	**144**	**12.78**	**48**	**0.1**	**0.88**
B: FTO/α-SnS/CdS/i-ZnO/ITO	200 ^{o}C, 40 min	95	7.91	43	0.1	0.32
**C: FTO/am-TiO_x/α-SnS/CdS/i-ZnO/ITO**	**As completed**	**85**	**3.74**	**31**	**0.1**	**0.1**
C: FTO/am-TiO_x/α-SnS/CdS/i-ZnO/ITO	200 ^{o}C, 20 min	-	-	-	-	Shunted
C: FTO/am-TiO_x/α-SnS/CdS/i-ZnO/ITO	200 ^{o}C, 40 min	-	-	-	-	Shunted
**π-SnS**						
**D: Mo/π-SnS/CdS/i-ZnO/ITO**	**As completed**	**133**	**5.93**	**27**	**0.1**	**0.21**
D: Mo/π-SnS/CdS/i-ZnO/ITO	150 ^{o}C, 20 min	-	-	-	-	Shunted
E: FTO/π-SnS/CdS/i-ZnO/ITO	As completed	180	2.00	40	0.1	0.14
E: FTO/π-SnS/CdS/i-ZnO/ITO	150 ^{o}C, 120 min	128	2.59	41	0.1	0.14
**E: FTO/π-SnS/CdS/i-ZnO/ITO**	**150 ^{o}C, 240 min**	**113**	**3.40**	**42**	**0.1**	**0.15**
F: FTO/am-TiOx/π-SnS/CdS/i-ZnO/ITO	As completed	180	2.50	32	0.1	0.14
F: FTO/am-TiOx/π-SnS/CdS/i-ZnO/ITO	150 ^{o}C, 120 min	200	3.50	31	0.1	0.22
**F: FTO/am-TiOx/π-SnS/CdS/i-ZnO/ITO**	**150 ^{o}C, 240 min**	**217**	**5.40**	**34**	**0.1**	**0.41**

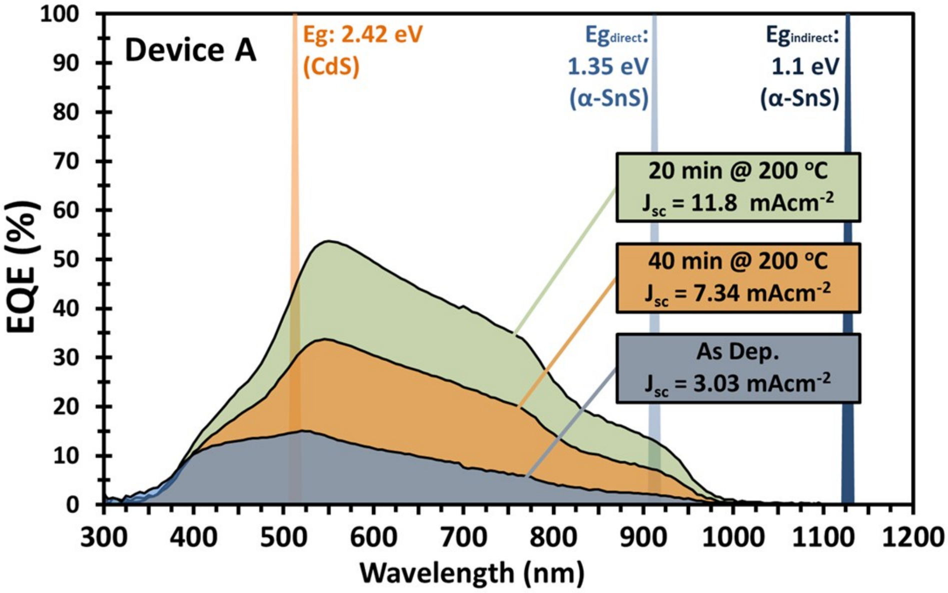

Fig. S2 External quantum efficiency (EQE) vs photon wavelength for device A consisting of Mo/α-SnS/CdS/ZnO/ITO. The EQE measurements after consecutive hot plate annealing treatments are presented.

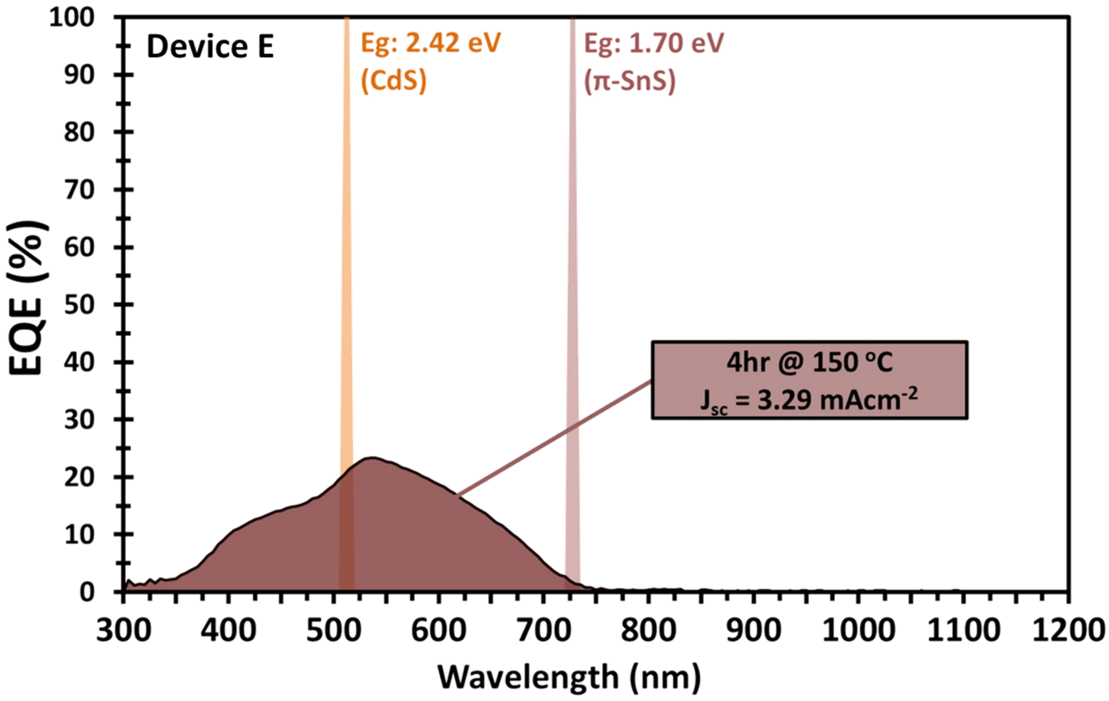

Fig. S3 The EQE vs photon wavelength for Device E after hotplate annealing at 150 ^{o}C for 4 hours

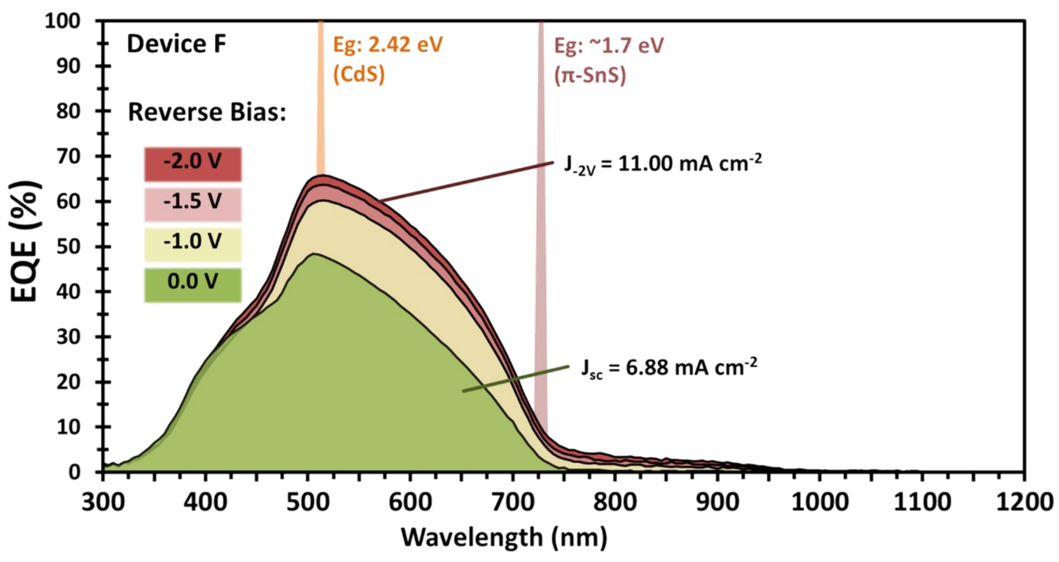

Fig. S4 The EQE vs photon wavelength for Device F after hotplate annealing at 150 °C for 4 hours at different reverse bias potentials from 0 V to -2 V.

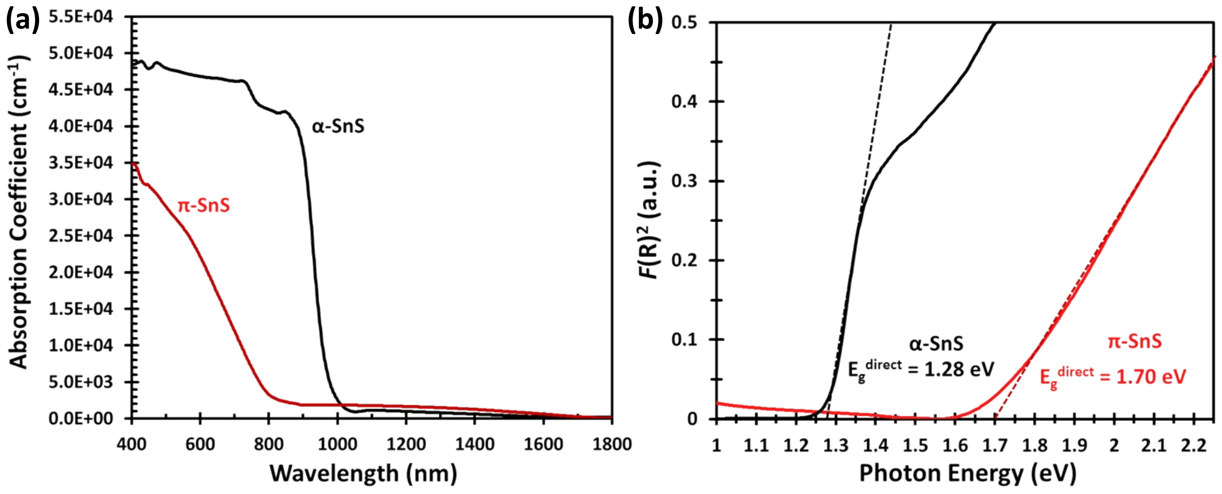

Fig. S5 (a) absorption coefficient vs. wavelength and (b) Tauc plot of the Kubelka–Munck function for direct allowed transitions, determined from the UV-Vis-NIR transreflectance measurements for α-SnS and π-SnS samples deposited on to glass.

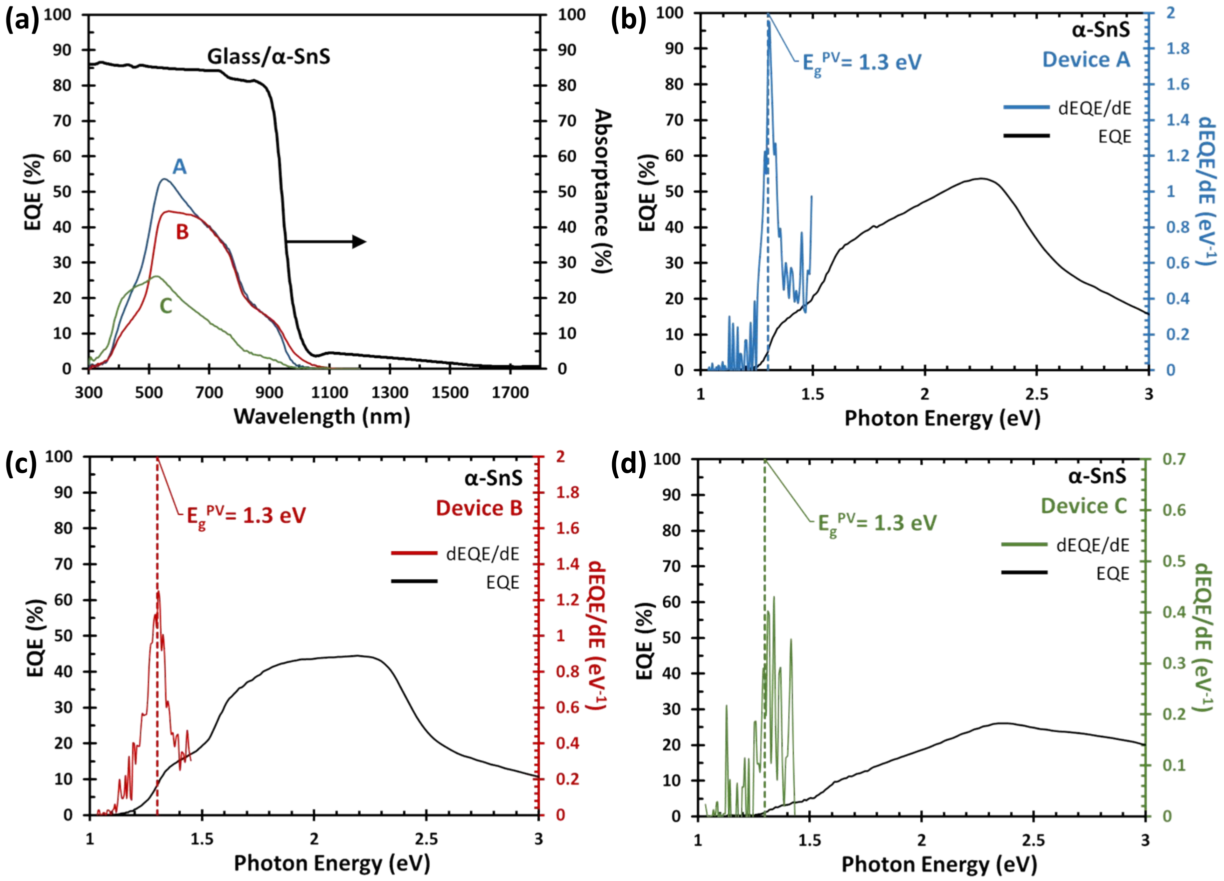

Fig. S6 (a) Combined absorptance data for α-SnS samples deposited onto glass (black) and the EQE data for optimized devices A (blue), B (red), and C (green) vs. wavelength. Measured EQE data (black) and its derivative (coloured) vs. photon energy, for devices consisting of α-SnS, (b)A, (c)B, and (d)C. The photovoltaic (PV) band gap (E_g^{PV}) is determined from the maximum point of the derivative, dEQE/dE.

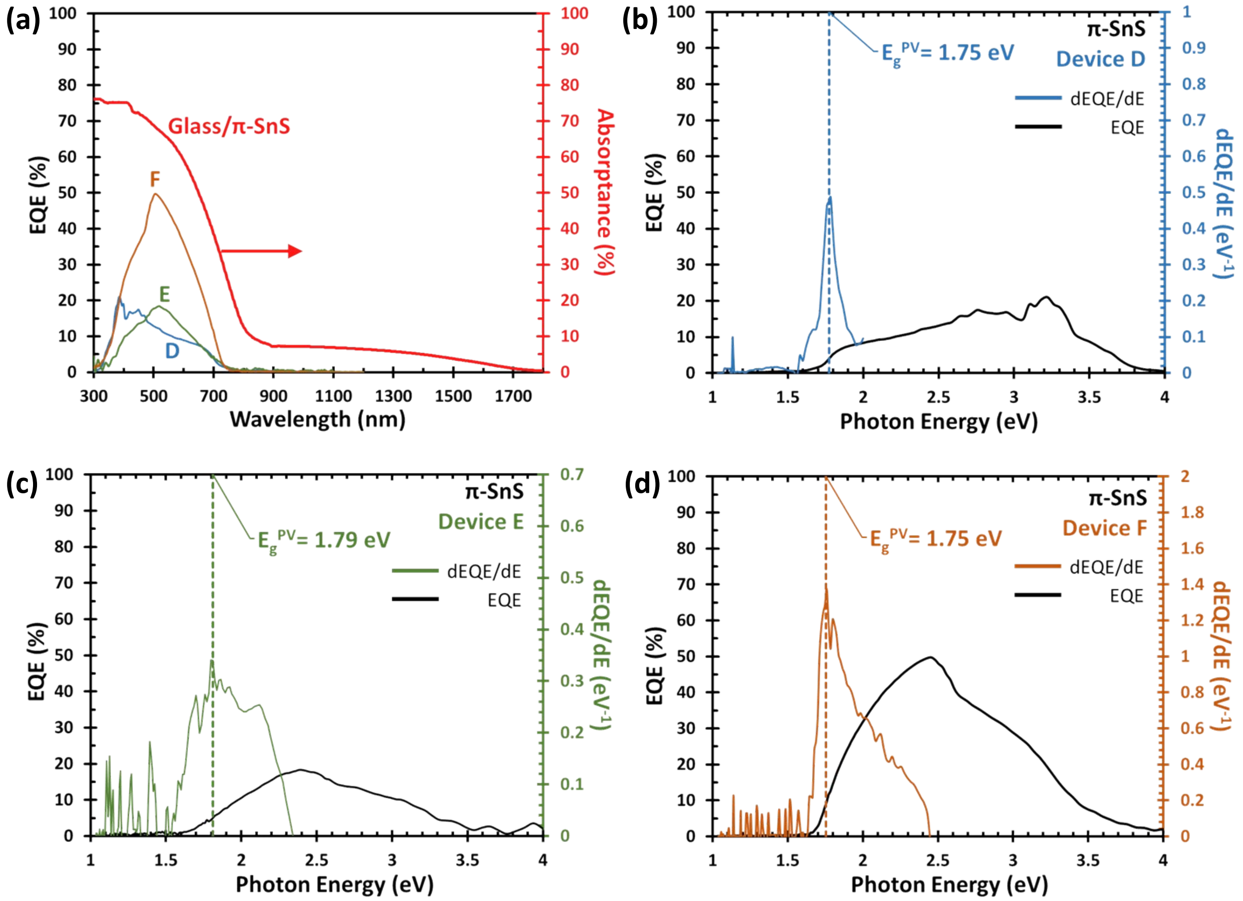

Fig. S7 (a) Combined absorptance data for π-SnS samples deposited onto glass (red) and the EQE data for optimized devices D (blue), E (green), and F (orange) vs. wavelength. Measured EQE data (black) and its derivative (coloured) vs. photon energy, for devices consisting of π-SnS, (b)D, (c)E, and (d)F. The photovoltaic (PV) band gap (E_g^{PV}) is determined from the maximum point of the derivative, dEQE/dE.

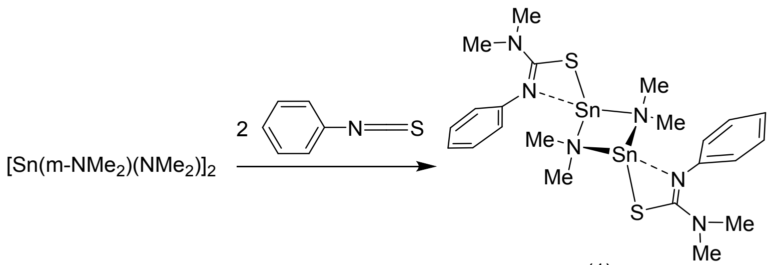

Scheme S1 Synthesis route to Dimethylamido-(N-Phenyl-N',N'-Dimethyl-Thiouriate)Sn(II) dimer [Sn({C_6H_5}NCSN{Me_2})(NMe_2)], the Sn(II) thio-ureide single source precursor (**1**).[1]

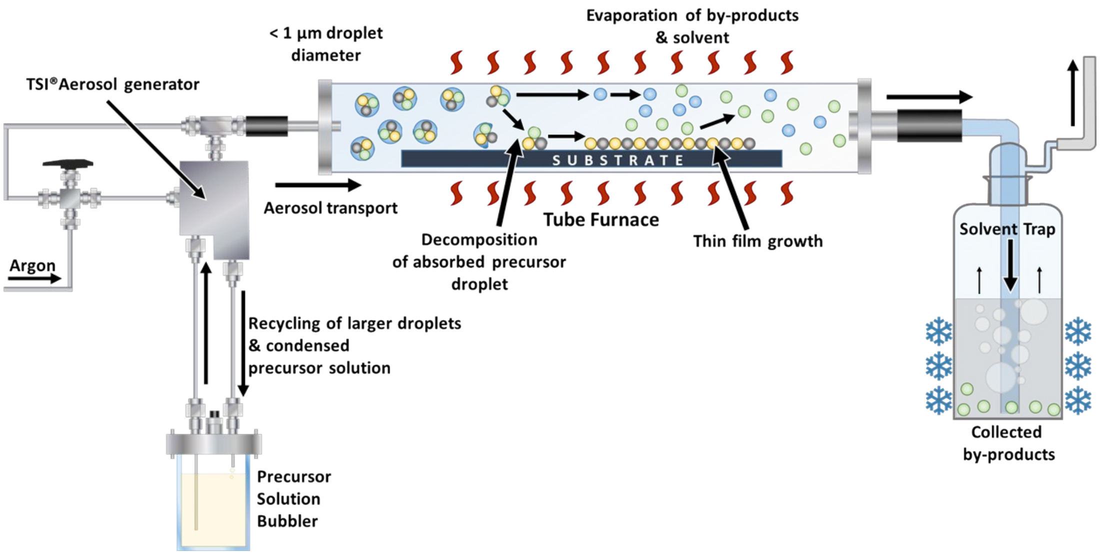

Fig. S8 Schematic of the aerosol assisted chemical vapor deposition (AA-CVD) apparatus used in this study. The aerosol was generated from a TSI® 3076 Aerosol generator.

References:

1. I. Y. Ahmet, M. S. Hill, A. L. Johnson and L. M. Peter, *Chemistry of Materials*, 2015, **27**, 7680-7688.
